# Measurements of Oxidative Potential of Particulate Matter at Belgrade Tunnel; Comparison of BPEAnit, DTT and DCFH Assays

**DOI:** 10.3390/ijerph16244906

**Published:** 2019-12-05

**Authors:** Maja V. Jovanovic, Jasmina Z. Savic, Farhad Salimi, Svetlana Stevanovic, Reece A. Brown, Milena Jovasevic-Stojanovic, Dragan Manojlovic, Alena Bartonova, Steven Bottle, Zoran D. Ristovski

**Affiliations:** 1 Vinca Institute of Nuclear Sciences, University of Belgrade, P.O. Box 522, 11001 Belgrade, Serbia; majaj@vin.bg.ac.rs (M.V.J.); jasnas@vin.bg.ac.rs (J.Z.S.); mjovst@vin.bg.ac.rs (M.J.-S.); 2 University Centre for Rural Health–North Coast, School of Public Health, University of Sydney, Sydney, NSW 2006, Australia; farhad.salimi@sydney.edu.au; 3 Centre for Air Quality & Health Research and Evaluation (CAR), An NHMRC Centre of Research Excellence, Glebe, NSW 2037, Australia; 4 School of Engineering, Deakin University, Melbourne, VIC 3216, Australia; 5 ILAQH (International Laboratory of Air Quality and Health), Queensland University of Technology, 2 George St., Brisbane, QLD 4000, Australia; reece.brown@hdr.qut.edu.au (R.A.B.); z.ristovski@qut.edu.au (Z.D.R.); 6 Faculty of Chemistry, University of Belgrade, Studentski trg 12–16, 11000 Belgrade, Serbia; manojlo@chem.bg.ac.rs; 7 South Ural State University, Lenin prospect 76, 454080 Chelyabinsk, Russia; 8 NILU–Norwegian Institute for Air Research, P.O. Box 100, 2027 Kjeller, Norway; alena.bartonova@nilu.no; 9 School of Chemistry, Physics and Mechanical Engineering, Queensland University of Technology (QUT), Brisbane, QLD 4000, Australia; s.bottle@qut.edu.au

**Keywords:** reactive oxygen species, PM_2.5_, PM_10_, online and offline OP probes

## Abstract

To estimate the oxidative potential (OP) of particulate matter (PM), two commonly used cell-free, molecular probes were applied: dithiothreitol (DTT) and dichloro-dihydro-fluorescein diacetate (DCFH-DA), and their performance was compared with 9,10-bis (phenylethynyl) anthracene-nitroxide (BPEAnit). To the best of our knowledge, this is the first study in which the performance of the DTT and DCFH has been compared with the BPEAnit probe. The average concentrations of PM, organic carbon (OC) and elemental carbon (EC) for fine (PM_2.5_) and coarse (PM_10_) particles were determined. The results were 44.8 ± 13.7, 9.8 ± 5.1 and 9.3 ± 4.8 µg·m^−3^ for PM_2.5_ and 75.5 ± 25.1, 16.3 ± 8.7 and 11.8 ± 5.3 µg·m^−3^ for PM_10_, respectively, for PM, OC and EC. The water-soluble organic carbon (WSOC) fraction accounted for 42 ± 14% and 28 ± 9% of organic carbon in PM_2.5_ and PM_10_, respectively. The average volume normalized OP values for the three assays depended on both the sampling periods and the PM fractions. The OP^BPEAnit^ had its peak at 2 p.m.; in the afternoon, it was three times higher compared to the morning and late afternoon values. The DCFH and BPEAnit results were correlated (*r* = 0.64), while there was no good agreement between the BPEAnit and the DTT (*r* = 0.14). The total organic content of PM does not necessarily represent oxidative capacity and it shows varying correlation with the OP. With respect to the two PM fractions studied, the OP was mostly associated with smaller particles.

## 1. Introduction

Atmospheric particulate matter (PM) is a heterogeneous mixture of extremely small particles and liquid droplets that get into the air [1]. The physical properties and the chemical composition of PM vary depending on meteorological conditions and emission sources and may contain inorganic and organic compounds, transition metals etc. [2,3]. The most detrimental effect to health can be attributed to the presence of respirable particles (RP). It is more prominent in accumulation mode (~80–1000 nm) due to the small size of particles and their large surface area, which enables them to have longer residence times in the air and deeper penetration in the lungs. In general, the exposure to PM is associated with respiratory and cardiovascular diseases, premature delivery, birth defects, low birth weight and premature death [4,5,6].

The chemical composition of PM and the determination of its oxidative potential (OP) representing the capacity of PM to oxidize target biomolecules are closely related to biological responses. The OP is an important metric used to assess the potential of PM to cause negative health effects [7,8]. Although the mechanisms of PM-related health effects remain incompletely understood, a leading hypothesis is that the exposure to PM leads to oxidative stress, induced by the formation of reactive oxygen species (ROS) within affected cells. ROS can be either exogenous (brought into the cell with PM) or endogenous (generated inside the cell upon exposure to PM). The presence of these species can damage important biological macromolecules, such as deoxyribonucleic acid (DNA) and ribonucleic acid (RNA), causing inflammation and cell apoptosis. At low ROS concentrations, cells are able to defend themselves against ROS damage through the assistance of antioxidant enzymes, while in the case that high levels of ROS are introduced to cells, intracellular defense mechanisms are not efficient and oxidative stress occurs [9]. OP can be determined either via direct measurement of radicals in the cells using chemical probes or exposure and examination of cells for oxidative stress markers (both animal and lab cultivated cells) [10].

For their simplicity, fast readouts and easy implementation, cell-free assays are much more practical for fieldwork and are frequently used in OP measurements [11,12]. Most commonly used cell-free, molecular probes are dithiothreitol (DTT), dichloro-dihydro-fluorescein diacetate (DCFH-DA) and ascorbic acid (AA). These probes differ in their sensitivity towards various organic and inorganic species that contribute to the overall OP as well as their stability and robustness [13]. There is no consensus on which cell-free assay for offline measurement of ROS is the most appropriate [14]. According to the available literature data, a high DTT activity is related to the presence of redox active species (metals, quinones, some water-soluble organic carbon (WSOC) and humic-like substances) [15] and low atmospheric dilutions (e.g., in the case of stagnant air inside a roadway tunnel where particle-phase semi-volatiles condense under such conditions) [16,17]. Semi-volatile compounds (for example, polycyclic aromatic hydrocarbons—PAHs), are oxidized to aromatic species, such as quinones, which have an influence on the ambient PM DTT activity [15]. Venkatachari and Hopke found that the DCFH assay was more nonspecific to ROS than either DTT or p-hydroxyphenylacetic acid assays [18]. The increasing preference for using DCFH as a probe is probably due to the fact that it can be oxidized non-discriminatorily by many groups such as RO_2_^·^, RO^·^, OH^.^, HOCl and ONOO^-^ [19]. The DCFH assay is simple and inexpensive, but it has some disadvantages that limit its application in real-time systems. The DCFH dye is unstable, it slowly oxidizes to fluorescent DCF in the air, and it is also photo-labile [19], which can generate false-positive results and higher background values that increase over time [20]. In contrast, 9,10-bis (phenylethynyl) anthracene-nitroxide (BPEAnit) has been reported to detect carbon and sulfur-centered free radicals as well as peroxyl and hydroxyl radicals [21], and it is stable for a long time towards light and auto-oxidation [22].

A variety of methods in both cell-free (offline and online) [23,24] and cell-based systems (in vivo and in vitro) are used to collect particles for subsequent ROS measurements [14,25,26]. In general, OP measurements depend heavily on the method used to collect particles for the OP analysis. Filter collection is a commonly used offline method, popular due to its excellent collection efficiency, practicality and low-cost. However, it is necessary to use organic solvents for particle extraction in order to achieve a good recovery of particles collected on the filters. Such a procedure can ultimately lead to biased results. Moreover, the aging of particles on the filter surfaces can cause underestimation (due to the evaporation of some organic species) or overestimation (if the particles become oxidized) of ROS, while the use of sonication for extraction can cause chemical changes and thermal degradation of the compounds present [27].

Numerous publications reported the OP of airborne PM in urban setting near sites exposed to the traffic, which was assessed by DTT [16,24,28] and DCFH assays [23,29,30,31]. There are available literature data with the comparison of different methods for OP estimation [32,33,34]. However, there are no literature data concerning the comparison between the OP values measured by online BPEAnit probe and the offline probes such as DTT and DCFH assays. The aim of this study was to investigate the OP of PM collected at the Terazije Tunnel with high traffic load, and at an urban background site. According to our knowledge, this is the first study to evaluate the comparative performance of common offline DTT and DCFH probes, and the online BPEAnit probe for the estimation of the OP. Whereas the offline measurements analyzed the OP content from particles collected on filters, the online measurements were performed by a modified Particle Into Liquid Sampler (PILS) and an average diurnal OP profile was developed. The diurnal OP profile was done to illustrate the magnitude of the real-time sampling effect and the scope of the information that can be gathered from online measurements. Finally, our study focused on the correlation between ROS and carbonaceous species.

## 2. Materials and Methods

The main aim of the study was to compare the results obtained by three probes for the OP assessment and to generate the data that may illustrate the probe properties, looking specifically for the contribution of vehicular traffic, a known source of substances with the high OP. Two sampling sites were selected: the first one with limited traffic contribution (the urban background, located at Vinca Institute, with an annual average PM_10_ concentration of approximately 26 µg·m^−3^), and the second one with high traffic contribution (the urban traffic site, located in vicinity of the Terazije tunnel, with an annual average PM_10_ concentration of approximately 45 µg·m^−3^). For offline assessment, the PM_2.5_ and PM_10_ samples were collected with low-volume samplers (LVS3, Sven Leckel, Germany) operated at each site for three 3-h periods (from 8 a.m. to 5 p.m.). Moreover, the collected filters were analyzed for elemental carbon (EC), organic carbon (OC) and water-soluble organic carbon (WSOC), and DTT and DCFH assays were used to assess the OP. Prior to the study, the sampling time and frequency were selected regarding to what considered the minimum quantity of PM that would lend itself to a successful assay analysis. For online assessment, a PILS with subsequent BPEAnit assay was employed at both sites from 8 a.m. to 5 p.m., obtaining hourly values. This online assay, as well as the filter collection, were carried out for 8 days at the urban traffic site and for 4 days at the background site. To make the results comparable, the measured hourly values obtained by PILS were presented as mean values of the three-hour measurements.

### 2.1. Sampling Locations

**Scheme 1:**
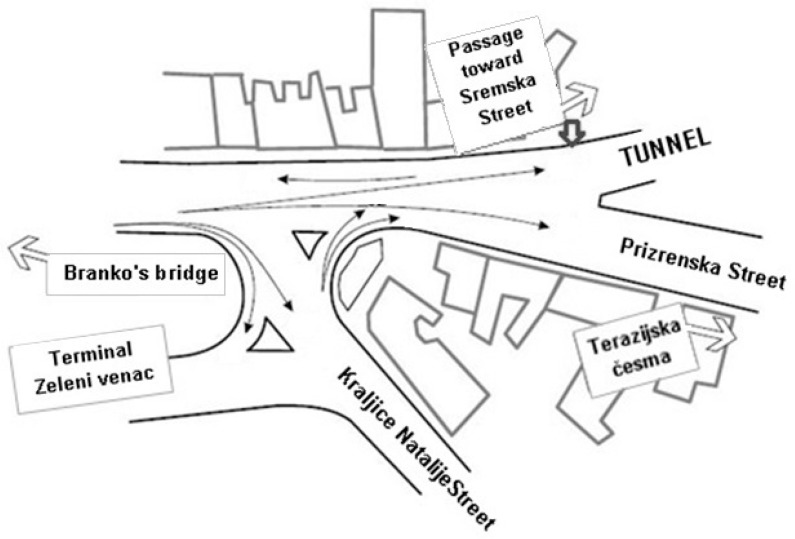
The sampling location.

The PM_10_ and PM_2.5_ samples were collected at two locations. The first location represented an urban site, at which the air pollution was highly influenced by vehicle emissions. This sampling site was located in Belgrade city center. The samplers were installed on a terrace above a pedestrian passage at the height of 1.5 m above ground, approximately 10 m away from the portal of the 223-meter-long Terazije Tunnel (see Scheme 1). The tunnel had the west and east portals with two-way traffic. Each direction had two lanes with no pavement. The average traffic density through the tunnel was 4000 vehicles per hour between 7 a.m. and 10 p.m., which was halved during the night. The second sampling site was an urban background 15 km away from the city center, at the Vinca Institute, and the samplers were set in a yard 1.5 m above ground.

### 2.2. PM Collection

The sampling campaign at the urban site was carried out from 18 until 29 May 2016, while at the urban background site it was done from 30th May until the 3rd of June 2016. Identical low-volume samplers (LVS3, Sven Leckel, Germany) with flow rates of 2.3 m^3^/h were used for collecting both fractions. The sampling was conducted in 3-h periods (between 8 a.m. and 11 a.m.; 11 a.m. and 2 p.m. and 2 p.m. and 5 p.m.). Quartz fiber filters (Whatman^®^ QA-M, 47 mm) were used for the collection of PM. Fifty-six sets of the filter samples were collected for both sites. All filters were pre-treated at 900 °C for three hours, to remove possible contamination, potentially present prior to the sampling. To minimize errors associated with sampling and analysis, three field blank filters were collected during this campaign. After the sampling (and before the analysis), all filters were kept in Petri dishes, wrapped in aluminum foil and frozen at −20 °C.

### 2.3. PM Mass Contraction

PM_10_ and PM_2.5_ were determined gravimetrically following SRPS EN 12341 standard (2005). Before weighing the filters both prior to and after the sampling, they were conditioned at a constant temperature (20 ± 1 °C) and relative humidity (50 ± 5%) during 48 h. All filters were weighed twice using a microbalance (MYA 5.3Y, RADWAG, reading precision of 1 µg).

### 2.4. Sample Preparation

The exposed and blank filters were cut into pieces of different shapes and dimensions for further analyses. For the determination of organic and elemental carbon, a punch area of 1.5 cm^2^ was used. The extraction procedure from one half of each filter was done with 12 mL of deionized water (18.2 MΩ), in falcon tubes using an orbital shaker (IKA^®^ KS 130) at 800 rpm for 90 min. This extraction procedure was used because sonication leads to ROS formation. It generates OH radicals, which can oxidize particles on the filter (or a solution) and increase the oxygenated organic aerosols (OOAs) fraction. This leads to positive artifacts in the reported measurements and can increase the reported ROS up to two orders of magnitude [27]. The obtained water extracts were filtered through a syringe filter (0.2 µm nylon membrane), and used for WSOC and DTT measurements. DCFH analysis was performed on the filters with the surface of 0.739 cm^2^, as described in the subsection OP measurements (DCFH-DA assay).

#### 2.4.1. Carbon Analysis

Carbonaceous compounds in PM fractions, OC and EC, were determined by Carbon Aerosol Analyzer (Sunset Laboratory Inc.), using the thermo-optical transmittance protocol and following the NIOSH 5040 method. In brief, a filter punch was placed inside a quartz oven and heated passing through four temperature ramps (from 0 °C to 870 °C) [35]. The first phase was done in helium atmosphere, while the second phase was performed in 2% oxygen/helium atmosphere. A He–Ne laser light passing through the filter allows continuous monitoring of filter transmittance. Laser transmittance was used to correct the pyrolytically-generated EC. The carbon evolved during both phases was measured by a flame ionization detector (FID) after the conversion to CH_4_ inside the methanator. The instrument calibration was achieved through the injection of a known volume of methane into the sample oven for each analysis. The calibration constant was checked by a sucrose inner standard at least once each day when the instrument was used.

#### 2.4.2. WSOC Analysis

The determination of WSOC was done by TOC-V_CPN_ (Shimadzu) organic carbon analyzer using the non-purgeable organic carbon (NPOC) method. The first step in each analysis was acidifying a sample by 2M HCl, in a syringe. High-purity air (the carrier gas) was bubbled through the sample to eliminate carbonate carbon and carbon dioxide [36]. During the second step, the sample was injected into an oven and catalytically oxidized to carbon dioxide at 680 °C. The sample combustion products were then delivered by the carrier gas to the cell of non-dispersive infrared (NDIR) gas analyzer, where carbon dioxide was detected. The WSOC concentrations in the extracted samples were determined using a calibration curve, constructed for a series of potassium hydrogen phthalate standard solutions (concentration range from 0.4 to 2.8 µg·mL^−1^). The injection volume both for the standard and for the analyzed solutions was 30 µL. Each measurement was done in triplicate. In order to reduce possible bias, the syringe and the sample line were washed before each analysis.

### 2.5. OP Measurements

#### 2.5.1. DTT Assay

In this study, a slightly modified procedure of Cho et al. [37] was used. The time-dependent DTT consumption was measured in 15 min time intervals (from 0 to 90 min). The water extracts with a known PM mass were incubated at 37 °C with DTT (100 µM) in 0.1 M potassium phosphate buffer at pH 7.4 (1 mL total volume). After the incubation, 100 µL of 10% trichloroacetic acid was added to stop the reaction. An aliquot of 0.5 mL of the reaction mixture was taken and then mixed with 1 mL of 0.4 M Tris-HCl (pH 8.9) containing 20 mM ethylene diamine tetra-acetic acid (EDTA). Finally, this reaction mixture was shaken for 30 s after the addition of 25 µL of 10 mM 5, 5′-dithiobis-2-nitrobenzoic acid (DTNB). The concentration of the formed 2-nitro-5-thiobenzoic acid (TNB) product was measured spectrophotometrically at 412 nm (Perkin Elmer Lambda 35 spectrophotometer). Since such a reaction is sensitive to light [38], the whole experiment was done in the dark hood. The blanks and samples were analyzed in duplicates. The rate of the DTT loss for each sample was determined as the slope of its sample regression line minus the blank slope (according to the Charrier and Anastasio [15]).

#### 2.5.2. DCFH-DA Assay

The preparation of DCFH-HRP reaction mixture: 2 mL of 1 mM DCFH-DA in ethanol was mixed with 8 mL of 0.01 M potassium hydroxide, and it was left at the room temperature for 30 min to hydrolyze. After this time, the hydrolyzate was added to 1990 mL of 25 mM potassium phosphate buffer (pH 7.2), which contained 25 mg horseradish peroxidase (HRP, 179.2 U·mg^−1^).

The sampled and blank filters were suspended into an appropriate volume (2–4 mL) of DCFH-HRP reaction mixture, depending on their particulate mass. The reaction mixture was then sonicated for 15 minutes, since it was established in previous studies [27] that such a procedure will not alter the chemical composition of PM. This was followed by the 15-min incubation at 37 °C. A microplate reader (1420 VICTOR2, Perkin Elmer Wallac) was used to measure fluorescence at 535 nm (excitation at 485 nm). The DCFH-DA assay was done for the series of H_2_O_2_ standard solutions (from 0.80 to 6.32 × 10^−7^ M) to obtain a calibration curve. All samples and blanks were analyzed in duplicate and the results were expressed as H_2_O_2_ equivalents.

#### 2.5.3. The Profluorescent BPEA Nitroxide Assay

In this study, a modified version of the PILS was used, based on the design presented by Orsini et al. [39]. The air (with or without particles) coming into the system was mixed with a flow of steam; it then exited from an expanded cone and entered a condensation chamber, where the mixture rapidly cooled down forming a temperature gradient. The steam temperature at the point of injection was at the maximum of 105 °C, while the temperature of the aerosol flow exiting the chamber did not exceed 40 °C. Due to supersaturated conditions, the particles grew into 1–2 µm water droplets and were captured into a BPEAnit assay inside a vortex collector. The 1 µM BPEAnit was continuously introduced at the flow rate of 0.28 mL min^-1^ to react with the grown, condensed particles. The exiting liquid was collected at the same flow rate and analyzed by an in-line fluorometer (USB2000+, Ocean Optics) with Spectra Suite software at 484.79 nm (excitation at 430 nm). A calibration curve was created from 10 nM to 200 nM concentrations of BPEAnit-Methyl (a representative fully fluorescent probe-adduct species) was used to convert the fluorescence intensity generated in the sample into units reflecting ROS activity as in previous studies [22,40]. All the liquid flows were delivered using a peristaltic pump. The flow rate of the system was set to 5 L·min^−1^. The collection was done in a wetted wall cyclone, with a DMSO solution of the BPEAnit probe being the collection liquid. The collection time for each sample was 15 min, while the collected volume was 6 mL. The sampling of particles was done every 60 min. The fluorescence of the previously collected sample was measured, while HEPA filter was placed at the inlet of the instrument in order to do a background correction for the gas phase without particles. These measurements were done in the same way as for the gas phase with particles both before and after each particle-phase sampling. The value corresponding to the contribution of particle-phase activity was obtained by subtracting the fluorescence values recorded for non-filtered and filtered air.

To obtain comparable results of the BPEAnit probe with the samples collected on quartz filters, the sampling and measuring by PILS was done between 8 a.m. and 6 p.m.

### 2.6. Statistical Analysis

The relationships between variables were estimated using linear regression. The regression analysis was used to evaluate the relationship of the OP to the OC and to the EC. This was done to see if the estimated OP values depended on the OC content or on the primary vehicle pollution, well represented by EC emissions. In addition, the regression analysis was used to compare the trends among the OP values measured by different chemical probes. The observations derived from each type of the OP test were plotted against each other (points) together with an estimated regression line (solid line), and a 95% confidence interval associated with the estimate (grey area).

## 3. Results and Discussion

### 3.1. OC, EC and TC Concentrations

**Figure 1:**
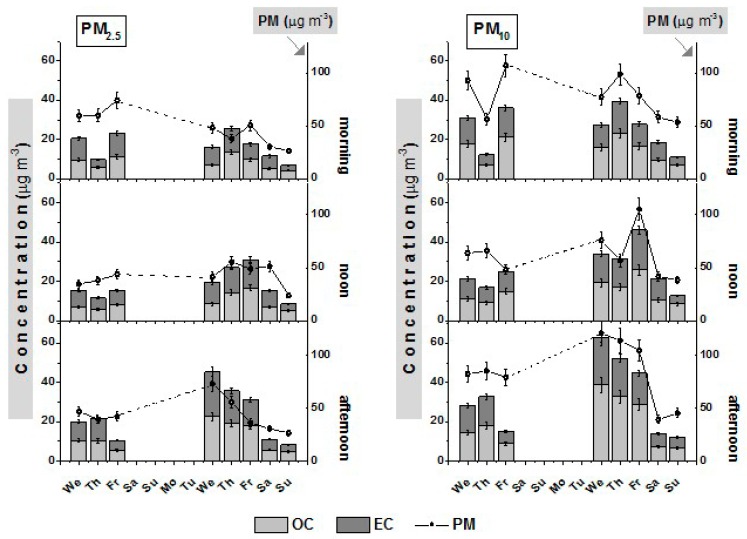
Daily dependence of OC (light grey color), EC (grey color) and PM mass concentrations (black circles and lines) of PM_2.5_ and PM_10_ in the morning, noon and afternoon, at the urban traffic site.

The OC, EC and TC concentrations were determined according to the procedure described in the subsection Sample preparation (Carbon analysis). The samples were taken at regular intervals, three times per day: in the morning, at noon and in the afternoon. The results for PM_2.5_ and PM_10_ and carbonaceous compounds are shown in Figure 1.

In the PM_2.5_ fraction, the values for OC and EC were approximately 30% lower in the morning than in the afternoon, although both sampling periods can be considered as the peaks in the traffic. These results can be explained by the deeper mixing of boundary layers in the afternoon. In addition, during the second week, the morning OC and EC values were 15% lower in comparison to the first week, while the noon and the afternoon values were approximately 50% higher. In contrast to EC, the OC values were either the same (morning and noon) or 10% higher (afternoon). In the PM_10_ fraction, the EC and OC concentrations were similar in the morning and at noon, while the afternoon values were slightly higher. During the second week, the morning OC and EC values were 6% lower, and approximately 40% higher at noon and in the afternoon, in comparison with the corresponding periods in the first week. It was also observed that the OC concentrations in PM_10_ fraction were, in most of the cases, 25–50% higher than the EC, which was more prominent in the second week of the sampling. The ratios of the PM_2.5_ and PM_10_ values for OC and EC were approximately 60% and 80%, respectively.

Table S1 (in Supplementary Material) shows that the OC concentrations varied between 4.37 µg·m^−3^ and 22.52 µg·m^−3^ in the PM_2.5_ fraction, while they were slightly higher, between 6.89 µg·m^−3^ and 38.77 µg·m^−3^, in the PM_10_. The EC concentrations ranged from 2.59 µg·m^−3^ to 22.80 µg·m^−3^ in PM_2.5_ and from 3.96 µg·m^−3^ to 23.89 µg·m^−3^ in PM_10_. The TC and the mass concentration trends were in good agreement for both PM_2.5_ and PM_10_. At the background site, as expected, the concentrations for OC, EC and TC were much lower than at the urban traffic site although they showed the same trends.

In Table S2, the results obtained in this study were compared with the available literature data. The reported OC and EC concentrations varied depending on the length of the tunnel, traffic flow, types of motor vehicles and ventilation in the tunnel. In most studies, the average OC and EC concentrations were higher than the values obtained in this study. However, taking into account that the Terazije Tunnel is shorter than the other tunnels presented in Table S2, the average OC and EC concentrations are considered high and comparable with the values recorded for the longer tunnels with more intense traffic flow. A possible reason for higher OC and EC values lies in the fact that the Terazije Tunnel is an urban tunnel with heavy traffic flow, especially during rush hour. Additionally, a huge number of diesel vehicles without any after-treatment devices (pre-European emission standard-IV) are still present in Serbia.

The concentrations of the WSOC fraction in PM_2.5_ for all sampling periods were in the range from 3.23 µg·m^−3^ to 4.72 µg·m^−3^, while the average WSOC/OC ratio was 0.42 ± 0.14. In the PM_10_ fraction, the WSOC concentrations ranged from 2.69 µg·m^−3^ to 6.56 µg·m^−3^, while the average WSOC/OC ratio was 0.28 ± 0.09.

### 3.2. OP^DTT^ and OP^DCFH^ Measurements and Their Correlation with PM Components (OC, EC and WSOC)

As described in the subsection OP measurements, the OP of the soluble fraction of PM was measured by DTT and DCFH assays. These results were normalized per PM mass and then correlated with the OC, EC and WSOC. Firstly, it can be observed that the OP at the busy tunnel site, measured by both the DTT and DCFH (OP^DTT^ and OP^DCFH^), was of similar intensity in both fractions. This could indicate that the OP was carried by the species found on particles within a very large range of sizes, which was particularly pronounced for the DCFH assay [32].

At the urban background site Vinca, the OP detected by the DTT, normalized per mass of PM, was similar in the PM_2.5_ and PM_10_ phases, but as PM_10_ contained a larger mass in comparison to PM_2.5_, this indicates that most of the OP is associated with the PM_2.5_ phase. Simonetti et al. [32] have already reported that the DTT assay is more sensitive to particles belonging to a fine mode. Moreover, Janssen et al. [24] obtained similar results for an urban background site. The ranges of OP^DTT^ and OP^DCFH^ values normalized per PM mass and per volume of air, as well as the average values with standard deviations are shown in Table 1. The difference between OP^DTT^ in both fractions as well as between OP^DCFH^, were analyzed for its statistical significance with Student’s *t*-test. For both assays (DTT and DCFH) there was a statistically significant difference between PM_2.5_ and PM_10_ fractions (*p* < 0.05).

Both OP methods showed almost the same inter-day variability. The results obtained by the DTT assay were generally quite low but in agreement with several previous studies [41,42,43]. The OP^DCFH^ were in the same range as in the studies of See et al., Khurshid et al. and Perrone et al. [23,30,44]. Similarly, the results recorded for OP^DTT^ were in line with previous studies [42,45]. Comparison of OP^DTT^ and OP^DCFH^ obtained in this study with literature data can be seen in Tables S3 and S4 in the Supplement. The higher values obtained for OP^DCFH^ in comparison to OP^DTT^ indicated that both of these assays were sensitive to different PM composition and could be dependent on a number of factors. One of the possible explanations for these results can be attributed to the fact that some potential species, which can contribute to the OP, remained in a residual fraction that was not examined in the DTT assay. Moreover, the DTT activity in the water-soluble fraction mostly depends on the metal content and organic species from combustion [46]. According to the literature, there are references that deal with the issue of soluble/insoluble part of PM, different extraction procedures as well as the correlation between soluble and insoluble fraction [47,48,49,50].

In addition, offline measurements by DCFH predominantly depend on ozone concentrations, temperature, solar radiation, sulfate, nitrate and certain transition metals [23,44]. Finally, several studies also found a good correlation with OC for both assays [37,44,51,52].

In Figures S1–S3 in the Supplement, DTT and DCFH measurements were presented as the amount of probe reacted, not normalized per mass of PM and then correlated with the mass of OC, EC and WSOC of corresponding samples. It can be observed that the OP did not correlate with the OC at the background site, while this correlation was modest at the busy tunnel site. Furthermore, a closer observation of the OC values reveals that one part of the OC mass that was contained in PM_10_ fraction did not have OP measurable by either DTT or DCFH. The measured OC values were extremely low at the background site, especially in the PM_2.5_ fraction. This further confirms the findings that not all the organic species contribute to the OP and also that not all of them are equally reactive and potent [40]. The DTT and DCFH measured amounts were similar at both sites, although the concentration of EC was much higher near the tunnel. As mentioned before, EC did not contribute to the measured OP. However, if EC is used as a marker for the traffic, an increase in the EC-correlated OP is an indicator that traffic-related sources are contributing to the OP. At the background site, the samples with very low EC concentrations showed a high OP as measured by DTT, and no correlation with EC was noticed. This may indicate that the measured OP did not come from combustion sources at the background site. As it was shown that the correlation between the OC and OP was not particularly strong, it can be assumed that not all the species in the OC fraction carried a detectable OP.

Finally, WSOC did not contribute to the species originating from primary sources and fresh exhaust and containing a detectable OP, as shown in Figure S3. The sampled particles did not have enough time to be aged photochemically; thus, the correlation with WSOC was modest, as previously noticed by Verma et al. [53].

**Figure 2:**
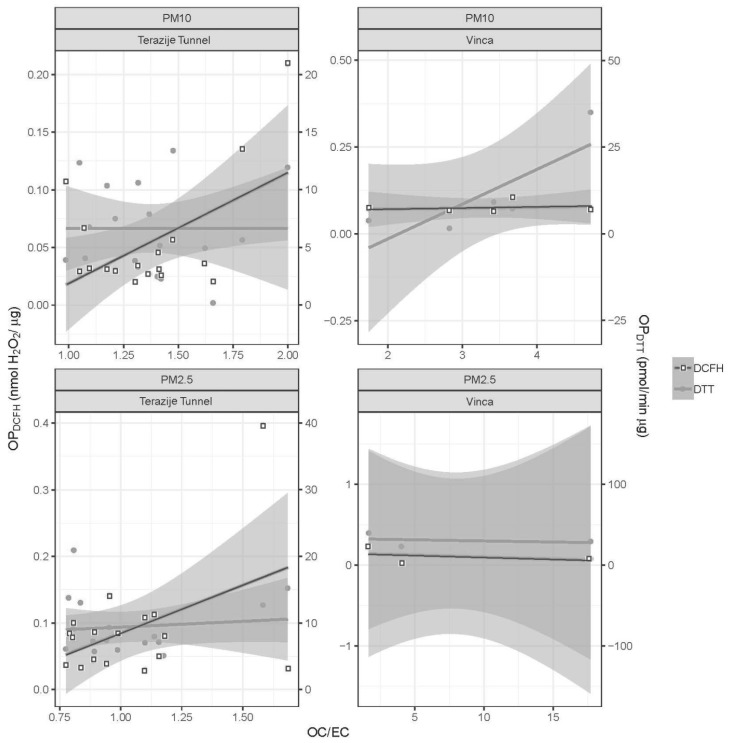
Association between OP measured by DTT (dithiothreitol) and DCFH (dichloro-dihydro-fluorescein) normalized per PM mass with OC/EC ratio.

In Figure 2, the OP, as measured by DCFH and DTT but normalized per unit mass, was presented as a function of the OC/EC ratio. The OC/EC ratio was used as a measure of the fraction of organic carbon in PM. It can be seen that the OP^DCFH^ rises with the increase of the OC/EC ratio, showing that the species that contributed to it were definitely organic. It can also be seen that the same did not apply to the DTT measurement, indicating that something else was the OP carrier. Some recent studies also report a lack of correlation between OC and DTT, citing the presence of traffic-related transition metals as a possible reason [44,54]. However, such presence was not observed at the background site. Finally, it can be concluded that these assays may detect different species on particles and that the information they provide is contradictory in many cases. To obtain a better insight into the potential toxicity of PM, DCFH and DTT results should be combined, providing a more holistic assessment.

### 3.3. Diurnal OP Profiles at the Busy Urban Site Measured by BPEAnit

The oxidative potential at the busy urban site was estimated by profluorescent BPEA nitroxide assay as previously mentioned in the subsection OP measurements (the profluorescent BPEA nitroxide assay).

**Figure 3:**
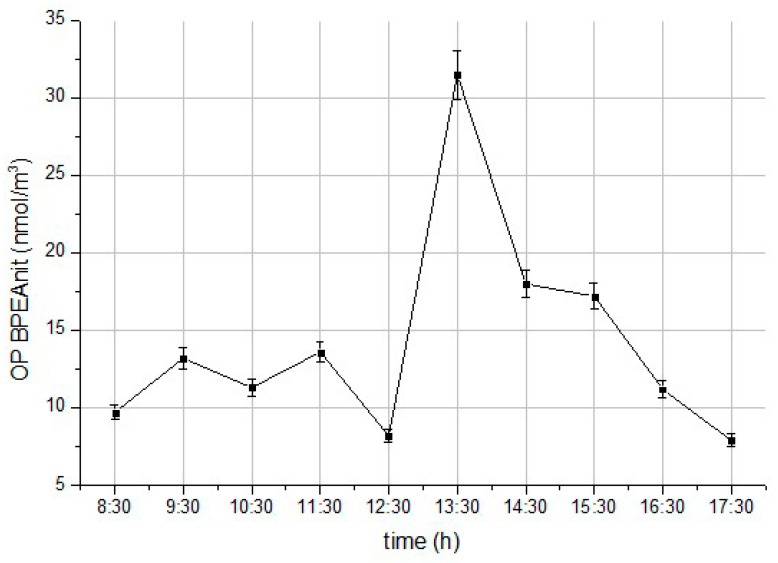
Average diurnal ROS (reactive oxygen species) profiles at the Terazije Tunnel site measured by BPEAnit (9,10-bis (phenylethynyl) anthracene-nitroxide).

Figure 3 shows diurnal profiles of the OP measured by BPEAnit in particle phase at the busy tunnel site in Belgrade city center. The OP was normalized per volume of air sampled, which represented the actual exposure better than the OP normalized per mass. The process was used to explore the nature of the ROS captured and its correlation with the sources and the sampling conditions. The OP was at its peak at the 2 p.m. rush hour. In the morning, it increased and remained more or less stable until 1 p.m., when it dropped by 30%. After the afternoon peak, the ROS concentration decreased steadily until 6 p.m. The OP measured during the afternoon rush hour was three times as high as the morning and late afternoon values. A diurnal OP profile illustrates the magnitude of the real-time sampling effect and the information that can be gathered from online measurements. Real-time systems allow us to detect the parts of the day when toxicity of the air is the highest and then to track the change throughout the day. Although PM-bound ROS cannot be used as a direct measure of toxicity, it can be used as a proxy for potential toxicity. Stevanovic et al. [22] measured PM-bound ROS of filtered (gas-phase) and non-filtered (neat) diesel and biodiesel exhaust and compared it to the results of direct human lung cells exposure. They found that both diesel exhaust significantly reduced cell viability and that the exposure resulted in a significant increase in inflammation, as showed by the increase in Interleukin 8 (IL-8) secretion and BPEAnit measurements. Real-time systems also facilitates our understating of OP values, i.e., their relationship with the meteorological factors, proximity of traffic or point sources, topography of the terrain, as well as the composition of primary emissions in general. As air is a dynamic system, only real-time monitoring can address these challenges.

**Figure 4:**
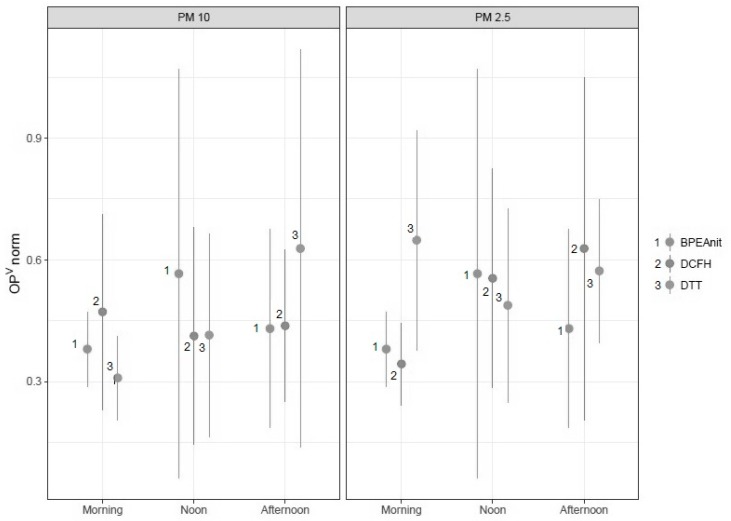
Comparison between the averages normalized OP values estimated by DTT, DCFH and BPEAnit for the Terazije Tunnel.

In order to have a better overview of the OP estimated by the three methods, the obtained results (expressed per volume of air) were normalized for all assays with respect to the highest value in each (OP^V^_norm_^DTT^, OP^V^_norm_^DCFH^ and OP^V^_norm_^BPEAnit^). The same procedure was applied for both PM fractions. In Figure 4, the average normalized OP values (OP^V^_norm_) were presented in a parallel manner on the scale from 0 to 1.

It is obvious that the average normalized OP values for the three assays depended on both the sampling period and the PM fraction. In the DTT assay, the OP^V^_norm_^DTT^ values were maximal in the morning for PM_2.5_ and in the afternoon for PM_10_. The OP^V^_norm_^DTT^ for PM_2.5_ at noon and in the afternoon were lower than in the morning rush hour by 24.8% and 11.7%, respectively. The OP^V^_norm_^DTT^ values for PM_10_ (in the morning and at noon) were, respectively, 50.9% and 34.1% lower than in the afternoon. In general, the DTT activity depends on the presence of redox active species and atmospheric dilution [15,16,17]. A possible reason for the maximal OP^V^_norm_^DTT^ (PM_2.5_) in the morning might lie in lower dispersion of particles, while for PM_10_ in the afternoon; it could be the presence of a high concentration of redox active species. The ratio of the average OP^V^_norm_^DTT^ values for two PM fractions (OP^V^_norm_^DTT^ PM_2.5_/ OP^V^_norm_^DTT^ PM_10_) was maximal in the morning (2.10 ± 0.65) and minimal in the afternoon (0.91 ± 0.36). On the contrary, the maximal values of the average normalized OP^DCFH^ (OP^V^_norm_^DCFH^) were in the afternoon (PM_2.5_) and in the morning (PM_10_). Compared to maximal OP^V^_norm_^DCFH^ values, the average normalized values for PM_2.5_ in the morning and noon were 45.4% and 11.7% lower, and for PM_10_ in the noon and afternoon 12.7% and 7.1% lower, respectively. The presence of many groups such as RO_2_^·^, RO^·^, OH^.^, HOCl and ONOO^-^ [19] contribute to the DCFH assay, while the DTT assay is more sensitive to redox active organic compounds (such as phenanthroquinone) or some transition metals (such as Cu, Mn and Fe) [15]. As shown in Figure 4, the average normalized values of DTT and DCFH during the three sampling periods were the opposite. This is also the direct confirmation that these probes are sensitive to different species.

**Figure 5:**
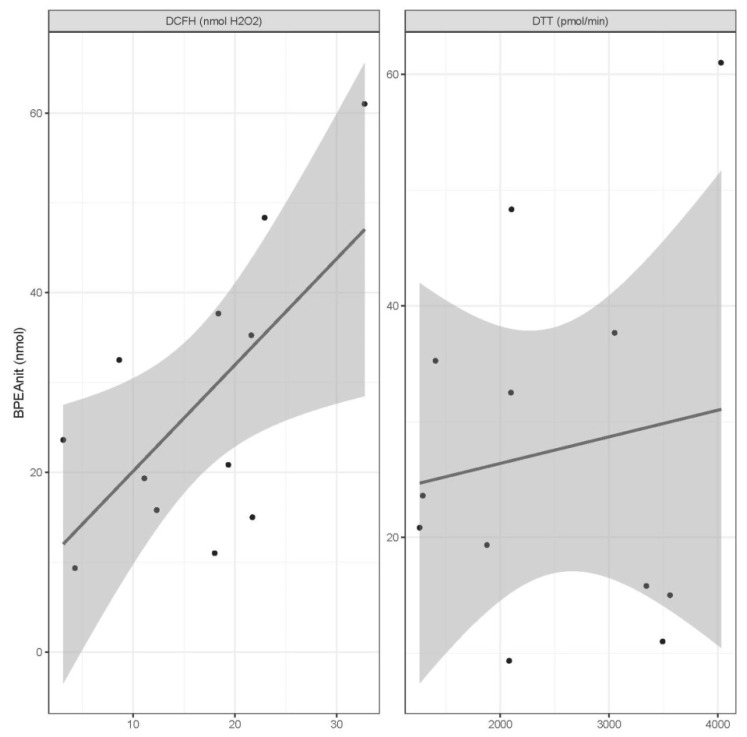
Association between OP measured by DTT and DCFH in PM_2.5_ fraction and BPEAnit for the Terazije Tunnel.

The ratio of the OP^V^_norm_^DCFH^ values for two PM fractions (OP^V^_norm_^DCFH^ PM_2.5_/ OP^V^_norm_^DCFH^ PM_10_) was maximal in the afternoon (1.43 ± 0.57) and minimal in the morning (0.73 ± 0.24). Finally, the average normalized OP value measured by BPEAnit was maximal at noon. Compared to the noon value, the average normalized OP^BPEAnit^ values (OP^V^_norm_^BPEAnit^) in the morning and in the afternoon were 32.9% and 23.8% lower, respectively. The maximal OP^V^_norm_^BPEAnit^ value at noon indicated that the concentration of peroxyl, hydroxyl, carbon and sulfur-centered free radicals were the highest during the noontime [21]. Besides, it can be seen in Figure 5 that the OP determined by DTT and DCFH assays was higher for smaller particles.

In order to compare the OP measured by the BPEAnit probe with the two other probes (DTT and DCFH), the BPEAnit readings were averaged over the same sampling period as for the other two probes. As can be seen from Figure 5, the correlation between the BPEAnit and the DCFH probes is moderately strong (*r* = 0.64), which is expected considering that these two probes detect similar species in the air (e.g., peroxy radicals) [13,19,55]. Additionally, the agreement between the OP trends measured by BPEAnit and DCFH is much better than between BPEAnit and DTT. On the contrary, the correlation between BPEAnit and DTT is modest (*r* = 0.14), and again related to the sensitivity of these probes and the difference in the sampling approach. The correlation between DTT and BPEAnit found in this study is much lower than previously reported in the study by Hedayat et al. [56], which can be attributed to the difference in the particle composition between real-world and laboratory-controlled emissions. In addition, Hedayat et al. [56] measured fresh emissions directly coming from one source (a diesel vehicle), while in this study the emissions from a number of different vehicles, both petrol and diesel, were thoroughly mixed before sampling. There is also the possibility of some atmospheric aging that could have further influenced this correlation. The advantage of BPEAnit in comparison to the other two assays is its sensitivity to a broad range of ROS and stability towards auto-oxidation and light. Furthermore, the sampling technique enables direct detection of PM-bounds ROS and also ROS contained in the gas-phase, which was shown to carry toxic potential comparable to the one found in the particle phase. In addition, DCFH and DTT assays require longer reaction time, even when applied in real-time measurements [57,58]. Both DTT and DCFH have shown association with various toxicological effects, while a similar association has not yet been investigated in the case of BPEAnit. For this reason, future research should be focused on more extensive investigation of the association of the ROS obtained by BPEAnit with potential health and toxicological effects. Appling several probes parallel provides more comprehensive insight into the toxicity of particles and artifacts of individual probes could be minimized. Although these methods cannot directly provide insight into the toxicological effects, they represent a good tool for predicting ROS formation and estimation of potential health risks. Since BPEAnit and DCFH assays measure the amount of ROS that is present on and/or within PM while DTT assay measures the ability of PM to generate ROS, they represent complementary methods.

## 4. Conclusions

In this study, the OP^DTT^ and OP^DCFH^ of two PM fractions were assessed and compared with the OP results obtained by BPEAnit probe. To the best of our knowledge, this is the first study that directly compares the performance of these offline probes with the online BPEAnit probe. The sampling was done in the proximity of a busy urban tunnel and at an urban background site.

The average concentrations of both PM fractions and their OC and EC concentrations were 44.8 ± 13.7, 9.8 ± 5.1 and 9.3 ± 4.8 µg·m^−3^ (PM_2.5_) and 75.5 ± 25.1, 16.3 ± 8.7 and 11.8 ± 5.3 µg·m^−3^ (PM_10_), respectively. The WSOC fraction accounted for 42 ± 14% and 28 ± 9% of OC in PM_2.5_ and PM_10_, respectively. It was observed that the total organic content of PM does not necessarily represent the oxidative capacity and that depending on the source, it can correlate with the OP in different ways. The correlation between the OP^DCFH^ and the organic content was higher than the corresponding correlation between the OP^DTT^ and the OC. Since the correlation between the OP and EC was positive, the traffic is attributed to be the main source of EC; however, EC was not the carrier of the OP. A strong relationship between the OP and WSOC was not observed. The average normalized OP values for three assays depended on both the sampling periods and PM fractions. Most of the detected OP was contained within the PM_2.5_ fraction. The OP^DCFH^ and OP^BPEAnit^ results demonstrated a moderately strong correlation (*r* = 0.64), while there was no good agreement between OPBPEAnit and OP^DTT^ (*r* = 0.14). Still, considerable work remains to be done in order to standardize the sampling methodology and the detection of a wide range of ROS species.

## Figures and Tables

**Table 1:** Minimal, maximal and average OP^DTT^ and OP^DCFH^ values normalized per PM mass and per volume of air.

Oxidative Potential	PM_2.5_			PM_10_		
	Min	Max	Average ± std	Min	Max	Average ± std
OP^DTT^ pmol DTT (min·µg)^−1^	5.06	20.90	9.43 ± 4.27	0.18	13.39	6.66 ± 3.90
OP^DCFH^ nmol H_2_O_2_ eq·µg^−1^	0.03	0.40	0.09 ± 0.09	0.02	0.21	0.05 ± 0.05
OP^DTT^ pmol DTT (min·m^3^)^−1^	153.03	638.17	365.67 ± 149.74	20.99	1059.72	457.82 ± 290.81
OP^DCFH^ nmol H_2_O_2_ eq·m^−3^	0.62	6.38	3.14 ± 1.65	0.83	7.25	3.20 ± 1.60

## Supplementary Materials

The following are available online at https://www.mdpi.com/1660-4601/16/24/4906/s1, Table S1: Maximal, minimal and average values of PM, OC, EC, and TC mass concentrations in Terazije Tunnel (PM_2.5_ and PM_10_). Percent of OC, EC and TC in overall PM mass, Table S2: Comparison of the average daily OC and EC concentrations with other tunnel studies, Table S3: Comparison of OP^DTT^ (expressed per mass and volume of air) with other studies, Table S4: Comparison of OP^DCFH^ (expressed as nmol H_2_O_2_ m^−3^) with other studies, Figure S1: Correlation between OP measured by DTT and DCFH and EC for fractions PM_2.5_ and PM_10_, Figure S2: Correlation between OP measured by DTT and DCFH and OC for fractions PM_2.5_ and PM_10_, Figure S3: Correlation between OP measured by DTT and DCFH and WSOC for fractions PM_2.5_ and PM_10_.
