# Supplementary material for: Measurements of Oxidative Potential of Particulate Matter at Belgrade Tunnel; Comparison of BPEAnit, DTT and DCFH Assays
Source: Int J Environ Res Public Health. 2019 Dec 5;16(24):4906. doi: 10.3390/ijerph16244906 (PMC6950172; doi:10.3390/ijerph16244906)
Supplement: Supplementary file 1 [file ijerph-16-04906-s001.doc]

**Measurements of Oxidative Potential of Particulate Matter at Belgrade Tunnel; Comparison of BPEAnit, DTT and DCFH Assays**

Maja V. Jovanovic ^1^, Jasmina Z. Savic ^1^, Farhad Salimi ^2,3^, Svetlana Stevanovic ^4, *^, Reece A. Brown ^5^, Milena Jovasevic-Stojanovic ^1^, Dragan Manojlovic ^6,7^, Alena Bartonova ^8^, Steven Bottle ^9^ and Zoran D. Ristovski ^5^,**^9^**

^1^ University of Belgrade, Vinca Institute of Nuclear Sciences, P.O. Box 522, 11001 Belgrade, Serbia;

^2^ University Centre for Rural Health–North Coast, School of Public Health, University of Sydney, Sydney 2006, Australia;

^3^ Centre for Air Quality & Health Research and Evaluation (CAR), An NHMRC Centre of Research Excellence, Glebe NSW 2037, Australia;

^4^ School of Engineering, Deakin University, VIC 3216, Australia;

^5^ ILAQH (International Laboratory of Air Quality and Health), Queensland University of Technology, 2 George St., Brisbane, 4000 QLD, Australia;

^6^ University of Belgrade, Faculty of Chemistry, Studentski trg 12–16, 11000 Belgrade, Serbia;

^7^ South Ural State University, Chelyabinsk, Lenin prospect 76, 454080, Russia; e-mail@e-mail.com

^8^ NILU–Norwegian Institute for Air Research, P. O. Box 100, 2027 Kjeller, Norway;

^9^ School of Chemistry, Physics and Mechanical Engineering, Queensland University of Technology (QUT), Brisbane, 4000 QLD, Australia;

**Supplementary Material**

**Table S1.** Maximal, minimal and average values of PM, OC, EC, and TC mass concentrations in Terazije Tunnel (PM_2.5_ and PM_10_). Percent of OC, EC and TC in overall PM mass.

|  | PM_2.5_ | | | | | | | PM_10_ | | | | | | |
| --- | --- | --- | --- | --- | --- | --- | --- | --- | --- | --- | --- | --- | --- | --- |
|  | Morning | | | | | | | | | | | | | |
|  | Concentration (µg/m^3^) | | | | % of overall PM mass | | | Concentration (µg/m^3^) | | | | % of overall PM mass | | |
|  | PM | OC | EC | TC | OC | EC | TC | PM | OC | EC | TC | OC | EC | TC |
| max | 74.18 | 13.51 | 12.29 | 25.80 | 35.56 | 32.33 | 67.89 | 107.08 | 23.21 | 16.38 | 39.59 | 23.44 | 16.54 | 39.98 |
| min | 26.45 | 4.37 | 2.59 | 6.96 | 10.01 | 6.60 | 16.61 | 53.61 | 7.10 | 3.96 | 11.06 | 12.61 | 7.38 | 20.62 |
| average | 48.47 | 8.39 | 8.12 | 16.52 | 18.12 | 17.29 | 35.41 | 77.89 | 14.83 | 10.70 | 25.53 | 18.33 | 13.26 | 31.59 |
| std | 16.22 | 3.23 | 3.63 | 6.65 | 7.55 | 7.75 | 14.72 | 20.58 | 6.20 | 4.43 | 10.56 | 3.87 | 3.21 | 6.82 |
|  | Noon | | | | | | | | | | | | | |
|  | Concentration (µg/m^3^) | | | | % of overall PM mass | | | Concentration (µg/m^3^) | | | | % of overall PM mass | | |
|  | PM | OC | EC | TC | OC | EC | TC | PM | OC | EC | TC | OC | EC | TC |
| max | 55.45 | 16.65 | 14.47 | 31.12 | 34.09 | 29.62 | 63.71 | 105.31 | 25.94 | 20.20 | 46.14 | 30.55 | 25.53 | 55.26 |
| min | 23.64 | 5.19 | 3.28 | 8.46 | 13.58 | 13.86 | 29.81 | 38.40 | 8.46 | 4.23 | 12.69 | 13.83 | 11.02 | 25.60 |
| average | 42.11 | 9.04 | 8.89 | 17.93 | 21.18 | 20.64 | 41.82 | 62.02 | 14.54 | 11.46 | 26.00 | 23.67 | 18.52 | 42.19 |
| std | 10.19 | 4.19 | 3.70 | 7.70 | 6.51 | 5.96 | 11.53 | 21.62 | 6.02 | 4.84 | 10.72 | 5.84 | 5.40 | 10.52 |
|  | Afternoon | | | | | | | | | | | | | |
|  | Concentration (µg/m^3^) | | | | % of overall PM mass | | | Concentration (µg/m^3^) | | | | % of overall PM mass | | |
|  | PM | OC | EC | TC | OC | EC | TC | PM | OC | EC | TC | OC | EC | TC |
| max | 72.91 | 22.52 | 22.80 | 45.32 | 49.41 | 36.96 | 86.37 | 120.73 | 38.77 | 23.89 | 62.65 | 32.11 | 19.79 | 51.90 |
| min | 26.45 | 4.91 | 3.41 | 8.33 | 13.33 | 11.70 | 25.03 | 39.15 | 6.89 | 5.22 | 12.11 | 11.21 | 7.59 | 18.80 |
| average | 43.73 | 12.06 | 10.92 | 22.98 | 26.73 | 23.69 | 50.41 | 83.72 | 19.43 | 13.22 | 32.65 | 21.46 | 15.29 | 36.75 |
| std | 14.88 | 6.88 | 6.59 | 13.36 | 11.47 | 9.33 | 20.38 | 29.75 | 12.43 | 6.81 | 19.06 | 7.29 | 3.90 | 10.58 |

**Table S2.** Comparison of the average daily OC and EC concentrations with other tunnel studies.

| PM size of fraction | OC (µg/m^3^) | EC (µg/m^3^) | Sampling location | Sampling duration | Length of tunnel (m) | Analyt. method | Protocol | Sampling place | Reference |
| --- | --- | --- | --- | --- | --- | --- | --- | --- | --- |
| ≤ 2.5 | 59.2 | 112.2 | Roadway tunnel, Marseille | 12 h | 2445 | TOT | NIOSH | Approximately  200 m from the exit | [1] |
| ≤ 10 | 79.3 | 129.0 |  | 0.5 h |  |  |  |  |  |
| ≤ 2.5 | 23.9 | 18.9 | Highway tunnel, Taiwan | Between 3-6.5 hours | 12 900 | TOR | IMPROVE | Inside the tunnel | [2] |
| ≤ 2.5 | 30.9 | 18.6 | Urban road tunnel, Wellington | 4 a.m. – 8 a.m. and 2 p.m. – 6 p.m. | 623 | TOR | NIOSH | A ventilation outlet on the eastern side of the tunnel | [3] |
|  | 21.7 | 21.3 |  |  |  |  | IMPROVE |  |  |
| ≤ 2.5 | 13.8 | 8.7 | Road tunnel, the Jânio Quadros (JQ), São Paulo | 8 a.m. – 8 p.m. | 1900 | TOT | NIOSH | Roughly at the midpoint of  both tunnels | [4] |
|  | 59.7 | 121.6 | Road tunnel, the Rodoanel (RA), São Paulo |  | 1700 |  |  |  |  |
| ≤ 2.5 | 20.8 | 6.2 | Urban underwater tunnel, Guangzhou | 24 h (4-hr intervals) | 1238.5 | TOT | NIOSH | 50 m from the inlet | [5] |
|  | 47.8 | 23.7 |  |  |  |  |  | 50 m from the outlet |  |
| ≤ 2.5 | 57.0 | 176.0 | Road tunnel, Portugal | 8 a.m. – 10 a.m. and 5 p.m. – 7 p.m. | 1725 | TOT | NIOSH | Approximately in the middle of the tunnel | [6] |
| ≤ 10 | 72.0 | 185.0 |  |  |  |  |  |  |  |
| ≤ 2.5 | 9.2 ± 1.2 | 9.2 ± 1.2 | Urban tunnel, Belgrade | 8 a.m. - 11 a.m., 11 a.m. - 2 p.m. and 2 p.m. - 5 p.m. | 223 | TOT | NIOSH | 10 m far from the tunnel | This study |
| ≤ 10 | 16.0 ± 2.1 | 11.7 ± 1.3 |  |  |  |  |  |  |  |

*TOT - Thermal optical transmittance; **TOR - Thermal optical reflectance.

**Table S3.** **Comparison of OP^DTT^ (expressed per mass and volume of air) with other studies.**

| PM size of fraction | OP^DTT^ | | Sampling duration | Sampling location and time | | | Note | Reference |
| --- | --- | --- | --- | --- | --- | --- | --- | --- |
|  | nmol DTT/(min µg) | nmol DTT/(min m^3^) |  |  |  |  |  |  |
| ≤ 2.5 | 0.083 | 1.32 | 5 h | Urban background (close apartment buildings and a school) | | March-October 2009 |  | [7] |
|  | 0.081 | 1.58 |  | Stop&go traffic (an inner-city intersection) | |  |  |  |
|  | 0.119 | 2.78 |  | Continuous traffic (the exit of a tunnel) | |  |  |  |
| ≤ 10 | 0.059 | 1.53 |  | Urban background (close apartment buildings and a school) | |  |  |  |
|  | 0.072 | 2.46 |  | Stop&go traffic (an inner-city intersection) | |  |  |  |
|  | 0.089 | 3.54 |  | Continuous traffic (the exit of a tunnel) | |  |  |  |
| ≤ 2.5 | 0.008 | / | / | Urban site (USC campus) and Claremont) | November 2001-March 2002 | |  | [8] |
| ≤ 10 | 0.003 | / |  |  |  |  |  |  |
| ≤ 2.5 | 0.013 | 0.48 | / | Urban site (University  Park Campus of USC) | | October– November 2007 |  | [9] |
| ≤ 2.5 | 0.028 | 0.35 |  | Urban site (central Los Angeles) | | July 2012–February 2013 |  | [10] |
| 0.25μm - 2.5μm | 0.017 | 0.16 | 24 h | Urban site ( USC campus) | | March-May 2007 |  | [11] |
| 2.5μm - 10μm | 0.012 | 0.10 |  |  |  |  |  |  |
| ≤ 2.5 | 0.036 | 0.63 | 6 - 7 h | Downey (urban sites at University) | | June 2003 |  | [12] |
|  | 0.021 | 0.92 |  | Downey (urban sites at University) | | July 2003 |  |  |
|  | 0.068 | 2.50 |  | Caldecott tunnel B1 | | September 2004 |  |  |
|  | 0.075 | 1.16 |  | Caldecott tunnel B2 | | September 2004 |  |  |
|  | 0.025 | 0.37 |  | CA-110 (traffic freeway) | | January 2005 |  |  |
| ≤ 10 | 0.087 | 2.51 |  | Caldecott tunnel B1 | | September 2004 | Summation of PM_2.5_ and 2.5-10 fraction |  |
|  | 0.107 | 1.18 |  | Caldecott tunnel B2 | | September 2004 |  |  |
|  | 0.042 | 0.51 |  | CA-110 (traffic freeway) | | January 2005 |  |  |
| ≤ 2.5 | 0.011 ± 0.006 | 0.41 ± 0.19 | 3h (8 a.m. - 11 a.m.) | Terazije tunnel | | 18th till 29th May 2016 |  | This study |
|  | 0.010 ± 0.003 | 0.31 ± 0.14 | 3h (11 a.m. - 2 p.m.) |  |  |  |  |  |
|  | 0.007 ± 0.001 | 0.36 ± 0.10 | 3h (2 p.m. - 5 p.m.) |  |  |  |  |  |
| ≤ 10 | 0.004 ± 0.002 | 0.33 ± 0.12 | 3h (8 a.m. - 11 a.m.) |  |  |  |  |  |
|  | 0.009 ± 0.003 | 0.44 ± 0.25 | 3h (11 a.m. - 2 p.m.) |  |  |  |  |  |
|  | 0.008 ± 0.005 | 0.67 ± 0.24 | 3h (2 p.m. - 5 p.m.) |  |  |  |  |  |

**Table S4.** Comparison of OP^DCFH^ (expressed as nmol H_2_O_2_ m^-3^) with other studies.

| Pm size of fraction | DCFH (nmol H_2_O_2_ m^-3^) | Sampling duration | Sampling location and time | | Note | Reference |
| --- | --- | --- | --- | --- | --- | --- |
| ≤ 2.5 | 1.25 | 3 h (between 10 a.m. – 3 p.m.) | Urban site (the University of Texas at Austin campus) | November 2011 and September 2012 |  | [13] |
|  |  |  |  |  |  |  |
| ≤ 3.2 | 0.54 | 3 h | Sidewalk | July – September 2000 | Summation of mean of 3 size fractions from 0.18 to 3.2 µm | [14] |
|  |  |  |  |  |  |  |
| 3.2μm - 10μm | 0.06 |  |  |  |  |  |
|  |  |  |  |  |  |  |
| ≤ 2.5 | 15.10 | 3 h (10 a.m. – 1 p.m.) | Traffic (a bus-stop, 2 m from the kerbside) | December 2005 |  | [15] |
|  |  |  |  |  |  |  |
| ≤ 2.5 | 0.85 | 3h (08-11 a.m.) | Urban site (the New York Supersite at Queens College) | January 12, 2004 - February 5, 2004 | Summation of mean of 10 size fractions from 0.010 to 2.5 µm | [16] |
|  | 0.97 | 3h (12-3 p.m.) |  |  |  |  |
|  | 0.87 | 3h (4-7 p.m.) |  |  |  |  |
| ≤ 10 | 0.15 | 3h (08-11 a.m.) |  |  | Summation of mean of 2 size fractions from 2.5 to 10 µm |  |
|  | 0.17 | 3h (12-3 p.m.) |  |  |  |  |
|  | 0.14 | 3h (4-7 p.m.) |  |  |  |  |
| ≤ 2.5 | 2.18 ± 0.70 | 3h (8 a.m. - 11 a.m.) | Terazije tunnel | 18th till 29th May 2016 |  | This study |
|  | 3.54 ± 1.64 | 3h (11 a.m. - 2 p.m.) |  |  |  |  |
|  | 4.00 ± 2.17 | 3h (2 p.m. - 5 p.m.) |  |  |  |  |
| ≤ 10 | 3.42 ± 1.88 | 3h (8 a.m. - 11 a.m.) |  |  |  |  |
|  | 2.98 ± 1.85 | 3h (11 a.m. - 2 p.m.) |  |  |  |  |
|  | 3.17± 1.09 | 3h (2 p.m. - 5 p.m.) |  |  |  |  |


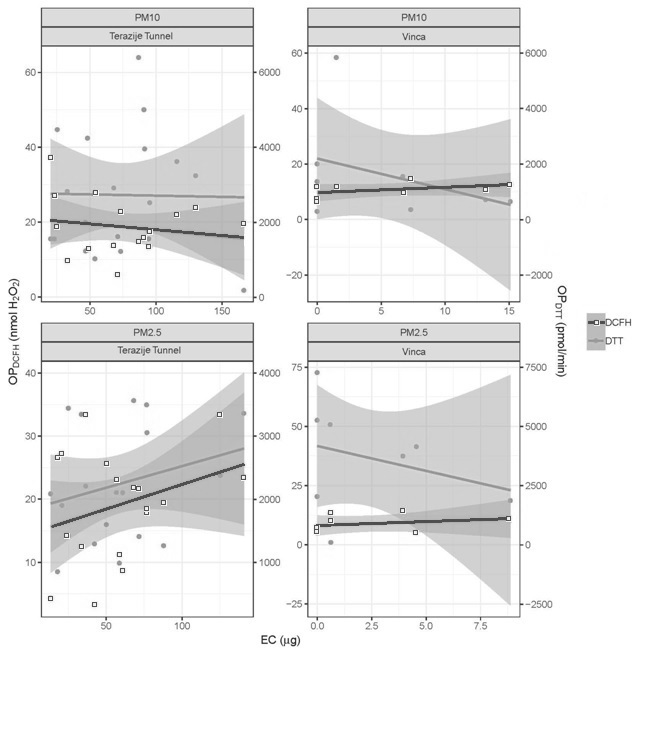


**Figure S1.** Correlation between OP measured by DTT and DCFH and EC for fractions PM_2.5_ and PM_10_.


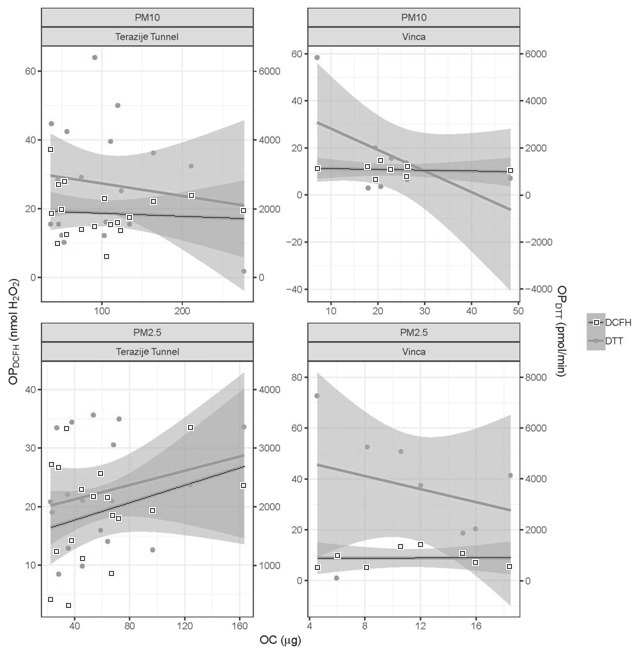


**Figure S2.** Correlation between OP measured by DTT and DCFH and OC for fractions PM_2.5_ and PM_10_.


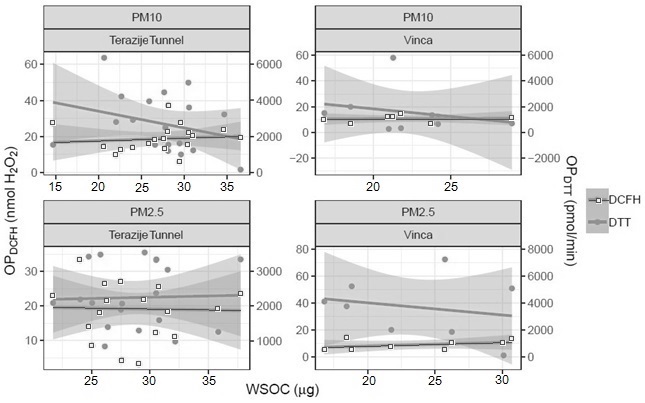


**Figure S3.** Correlation between OP measured by DTT and DCFH and WSOC for fractions PM_2.5_ and PM_10_.

**REFERENCES (Supplement)**
